# *Helicobacter pylori* geographic variation: a window into global differences in gastric cancer incidence

**DOI:** 10.1128/mmbr.00204-24

**Published:** 2026-07-27

**Authors:** Chiamaka D. Okoye, Georgia C. Caso, Timothy L. Cover

**Affiliations:** 1 Department of Pathology, Microbiology and Immunology, Vanderbilt University Medical Center 204907, Nashville, Tennessee, USA; 2 Department of Medicine, Vanderbilt University School of Medicine 12327, Nashville, Tennessee, USA; 3 Vanderbilt Institute for Infection, Immunology, and Inflammation, Vanderbilt University Medical Center 12328, Nashville, Tennessee, USA; 4 Veterans Affairs Tennessee Valley Healthcare System, Nashville, Tennessee, USA; University of Wisconsin-Madison, Madison, Wisconsin, USA

**Keywords:** *Helicobacter pylori*, gastric cancer, evolution, population genetics, pathogenesis

## Abstract

*Helicobacter pylori* infection is the strongest known risk factor for gastric cancer, a leading cause of cancer-related deaths worldwide. There is marked global geographic variation in both the incidence of gastric cancer and genetic features of *H. pylori* strains. Geographic diversity among *H. pylori* strains can be linked to ancestral human migrations and subsequent *H. pylori* evolution within human population groups. In this review, we discuss how geographic diversity among *H. pylori* strains is an important factor contributing to geographic variations in gastric cancer incidence. We focus on geographic variations in genes encoding secreted *H. pylori* proteins (CagA and VacA), outer membrane proteins (BabA, HopQ, HopH/OipA, HomB, AlpA, and AlpB), and enzymes required for lipopolysaccharide synthesis, and we discuss how these variations can impact gastric cancer pathogenesis. We discuss the concept of *H. pylori*-human co-evolution, including selective forces that may influence *H. pylori* evolution within human populations. Finally, we discuss recent changes in *H. pylori*–human relationships, including a declining prevalence of *H. pylori*, changes in *H. pylori* populations, and potential adverse consequences of mismatch between the ancestry of *H. pylori* strains and the ancestry of human hosts.

## *H. PYLORI* AS A RISK FACTOR FOR GASTRIC CANCER

About 13% of all human cancers worldwide (excluding non-melanoma skin cancer) are attributable to infectious agents (1). This corresponds to more than two million new cases of cancer attributable to infections each year (1). The most common infectious causes of cancer are *Helicobacter pylori*, papillomavirus, and hepatitis viruses, which are linked to gastric adenocarcinoma, cervix uteri carcinoma, and hepatocellular carcinoma, respectively. Epstein-Barr virus, human T-cell lymphotropic virus type 1 (HTLV-1), human herpesvirus type 8 (HHV-8), Merkel cell polyomavirus, and parasitic infections are additional infectious causes of cancer in humans.

*H. pylori* is a gram-negative bacterium that persistently colonizes the stomach in about half of the global population (2–4). *H. pylori* is typically acquired during childhood, and once acquired, it can persist for decades (5). The human stomach is the main reservoir for *H. pylori.* Person-to-person transmission has been documented within families, as well as among individuals who do not belong to the same family group (6, 7). The exact mechanisms of *H. pylori* transmission are not known. Fecal-oral transmission, transmission through saliva, and transmission through vomitus have all been proposed as possible mechanisms (8).

In many parts of the world, the prevalence of *H. pylori* is higher in older adults than in younger adults or children (9–11). This is attributed to a cohort effect, in which a high proportion of older adults were colonized with *H. pylori* during childhood. Decreased rates of *H. pylori* transmission over time are probably attributable to factors such as decreased family size, improved socioeconomic conditions, and increased antibiotic use (10, 11).

*H. pylori* colonization of the stomach triggers the development of a chronic gastric mucosal inflammatory response, termed non-atrophic gastritis (9). The gastric inflammatory response does not typically result in clearance of *H. pylori* and does not cause symptoms in most individuals. However, the presence of *H. pylori* is associated with an increased risk for the development of peptic ulcer disease and gastric cancer (12–14).

Gastric cancer is the fifth most common cause of cancer-related death worldwide (15–19). *H. pylori* is the strongest known risk factor for this malignancy (12–16). Other risk factors include male sex, dietary content (such as a high-salt diet, low-iron diet, or low intake of fruits and vegetables), environmental exposures (such as smoking), and other infectious agents (such as Epstein-Barr virus) (15, 20). Genome-wide association studies (GWAS) of European populations have identified multiple host genetic variations associated with an increased risk of non-cardia gastric cancer, including specific ABO blood group antigens (type A blood group) (21). Within a Japanese population, germline pathogenic variants in nine cancer-predisposing genes have been implicated in gastric cancer (20). In particular, pathogenic variants of genes involved in homologous recombination (*ATM*, *BRCA1, BRCA2*, and *PALB2*) conferred a strong additive risk for gastric cancer in *H. pylori*-positive patients (20). Polymorphisms in cytokine-encoding genes have also been associated with an increased risk of gastric cancer (22, 23). Several heritable syndromes are known to be risk factors for gastric cancer (e.g., hereditary diffuse gastric cancer arising from mutations in *CDH1* and familial cancer syndromes) (15, 24).

*H. pylori* colonization of the stomach is a risk factor for cancer of the distal stomach (non-cardia gastric adenocarcinoma), but not cancer of the proximal stomach (15). Of the two main types of gastric adenocarcinoma that can be differentiated histologically (intestinal-type and diffuse-type), *H. pylor*i is more closely associated with intestinal-type cancers (15). The development of *H. pylori*-associated intestinal-type gastric adenocarcinoma is preceded by a cascade of premalignant changes, including atrophic gastritis, intestinal metaplasia, and dysplasia (25). Experimental studies in animal models have provided strong evidence for an etiologic role of *H. pylori* in gastric cancer (26–33).

*H. pylori* is present in human populations throughout the world, but there is marked variation in *H. pylori* prevalence (34). *H. pylori* prevalence rates among adults in parts of Western Europe, North America, and Australia are estimated to be 20%–30%. In contrast, *H. pylori* prevalence rates are greater than 50% in many parts of the world, including Asia, Africa, Central America, and South America (34). The prevalence of *H. pylori* within a geographic region is potentially an important determinant of the gastric cancer rate in that region, but there is not a linear correlation (35).

Over the past century, there have been marked changes in the incidence of gastric cancer. In the early 1900s, gastric cancer was the most common cause of cancer-related death in the United States and many parts of Europe, but the incidence of gastric cancer has steadily decreased in these parts of the world over the past hundred years (15, 36). These changes in gastric cancer incidence over time can be attributed to multiple factors, including a decreasing prevalence of *H. pylori*, changes in the age of *H. pylori* acquisition, changes in diet or food storage, and increased antibiotic use. In contrast to the long-term declining incidence of distal gastric cancers associated with *H. pylori* (15, 36), an increased incidence of proximal (cardia) gastric cancers has been noted in the United States over the past few decades (37–39); these types of gastric cancers are not associated with *H. pylori*.

**Fig 1:**
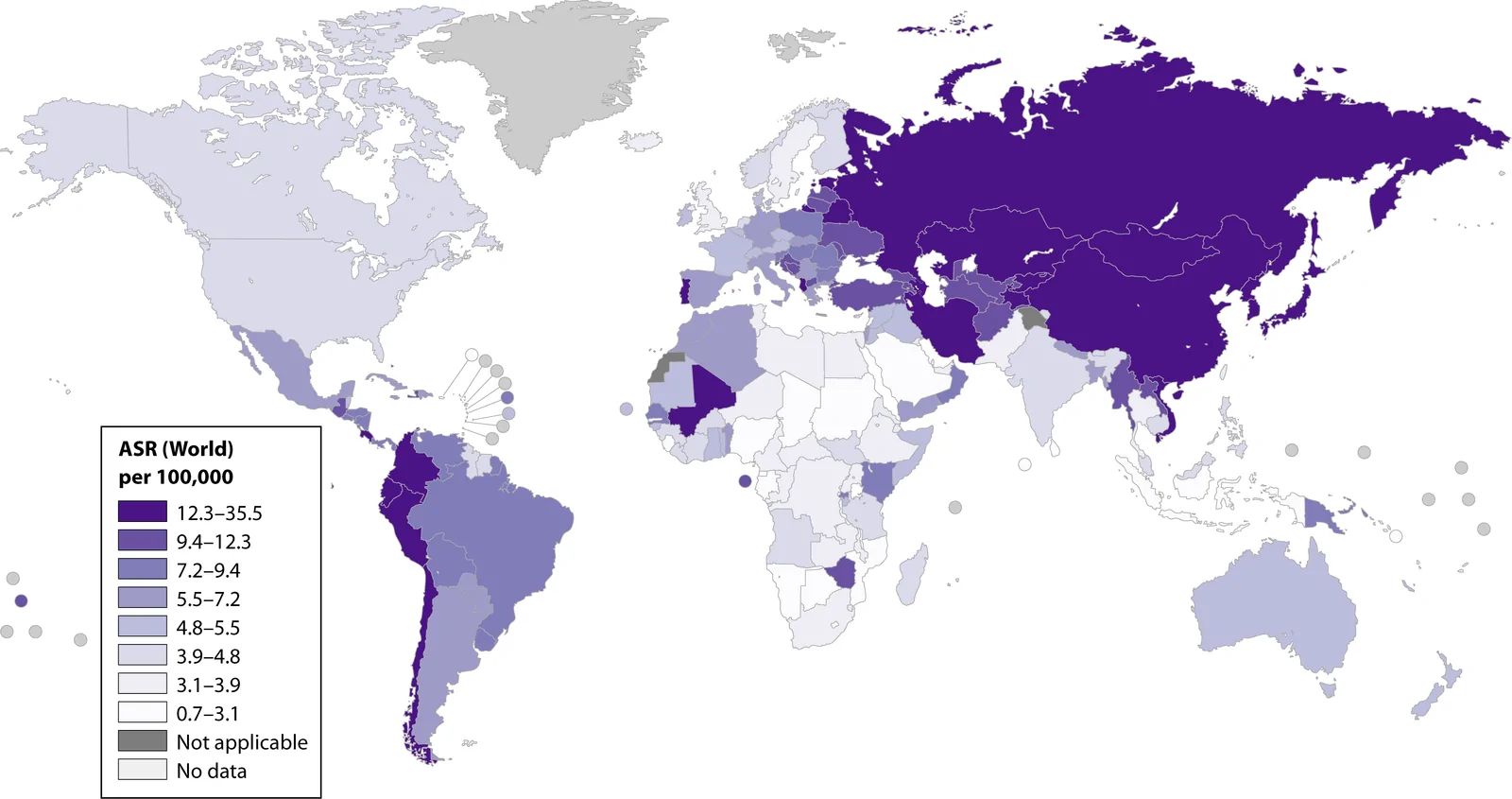
Global variation in gastric cancer incidence rates. The figure shows 2022 age-standardized rates (ASR) of gastric cancer incidence per 100,000 population (both sexes) (17). ©IARC/WHO All Rights Reserved 2026. Reproduced from https://gco.iarc.who.int/today/en/dataviz/maps-heatmap?mode=population&map_nb_color=8&cancers=7&palette=Purples (accessed 12 August 2025), with permission from IARC/WHO.

Long before the discovery of *H. pylori*, striking geographic variations in the incidence of gastric cancer were recognized (40), and these geographic variations are still observed in the 21st century. For example, gastric cancer rates are markedly higher in East Asia, parts of South and Central America, and Eastern Europe than in North America, Northern Europe, and most parts of Africa (Fig. 1) (17). There are many potential explanations for geographic variations in the incidence of gastric cancer, including geographic variations in host genetic characteristics, diet, environmental exposures, and *H. pylori* prevalence. In this review, we discuss geographic diversity in the genetic characteristics of *H. pylori* strains and the potentially important role of *H. pylori* genetic diversity as a factor contributing to geographic variations in gastric cancer incidence.

## *H. PYLORI* GENETIC VARIATION

### Overview of *H. pylori* genetic diversity

*H. pylori* strains from unrelated human hosts exhibit a high level of genetic diversity compared to strains of most other bacterial species (41–44). A core genome consisting of about 1,200 genes is present in all *H. pylori* strains (45, 46). Each strain also contains numerous strain-specific accessory genes (45–48), resulting in substantial variation in the gene content of unrelated strains. Strains isolated from unrelated human hosts exhibit differences in the nucleotide sequences of nearly all genes comprising the core genome (46, 49).

The sequence diversity of *H. pylori* genes is attributable to both a high mutation rate and a high rate of recombination (41–44, 50). *H. pylori* lacks several DNA mismatch repair proteins found in other bacterial species (51, 52), and a lack of DNA polymerase I proofreading activity further contributes to a high mutation rate (53). *H. pylori* undergoes intragenomic recombination and is naturally competent for uptake of exogenous DNA through the ComB type IV secretion system (54–58). Individual humans can be simultaneously colonized by multiple *H. pylori* strains (59), which allows uptake and acquisition of variant DNA sequences (60–62). Mutation, recombination, and genetic exchange occur over long periods of time in individual hosts (7, 60, 62–67).

To evaluate whether genetic variations among *H. pylori* strains influence the development of gastric cancer or peptic ulceration, numerous studies have compared the genetic characteristics of *H. pylori* strains isolated from patients with these diseases to corresponding genetic characteristics of strains isolated from patients with gastritis only (68, 69). Most studies have analyzed small numbers of genes, focusing on genes known to have important roles in *H. pylori*–host interactions, and most studies have been conducted using strains isolated from patients within restricted geographic regions. These studies have identified variations in *H. pylori* genes encoding secreted proteins and outer membrane proteins that are linked to disease outcomes (described in detail in subsequent sections) (68, 69). A GWAS of whole-genome sequences from 173 European *H. pylori* strains (including 49 isolated from patients with gastric cancer) confirmed that specific genetic variations relevant for *H. pylori*–host interactions (including the presence of *babA*, which encodes an outer membrane protein adhesin, and the presence of the *cag* pathogenicity island) are associated with gastric cancer (70). GWAS of Asian *H. pylori* strains (isolated from patients in Japan, Vietnam, Mongolia, or China) identified single-nucleotide polymorphisms (SNPs) or genes that differentiated gastric cancer isolates from non-gastric cancer isolates (71, 72). These include SNPs in *babB* (which encodes an outer membrane protein), *fecA2* and *frpB2* (which encode genes predicted to be involved in iron transport), *hemC* (involved in heme biosynthesis), *tlpC* (which encodes a chemoreceptor), *ftsY* (which encodes a protein required for cell division), and genes of unknown function. In addition, SNPs in numerous *H. pylori* genes contributing to metabolic functions have been linked to the development of gastric cancer or intestinal metaplasia, compared to less severe gastric histologic alterations (non-atrophic or atrophic gastritis) (73). The functional significance of most of the disease-associated SNPs identified in these GWAS has not been experimentally evaluated, and most have not been identified in multiple studies (71, 72, 74).

### Reconstructing the evolutionary history of *H. pylori*

**Fig 2:**
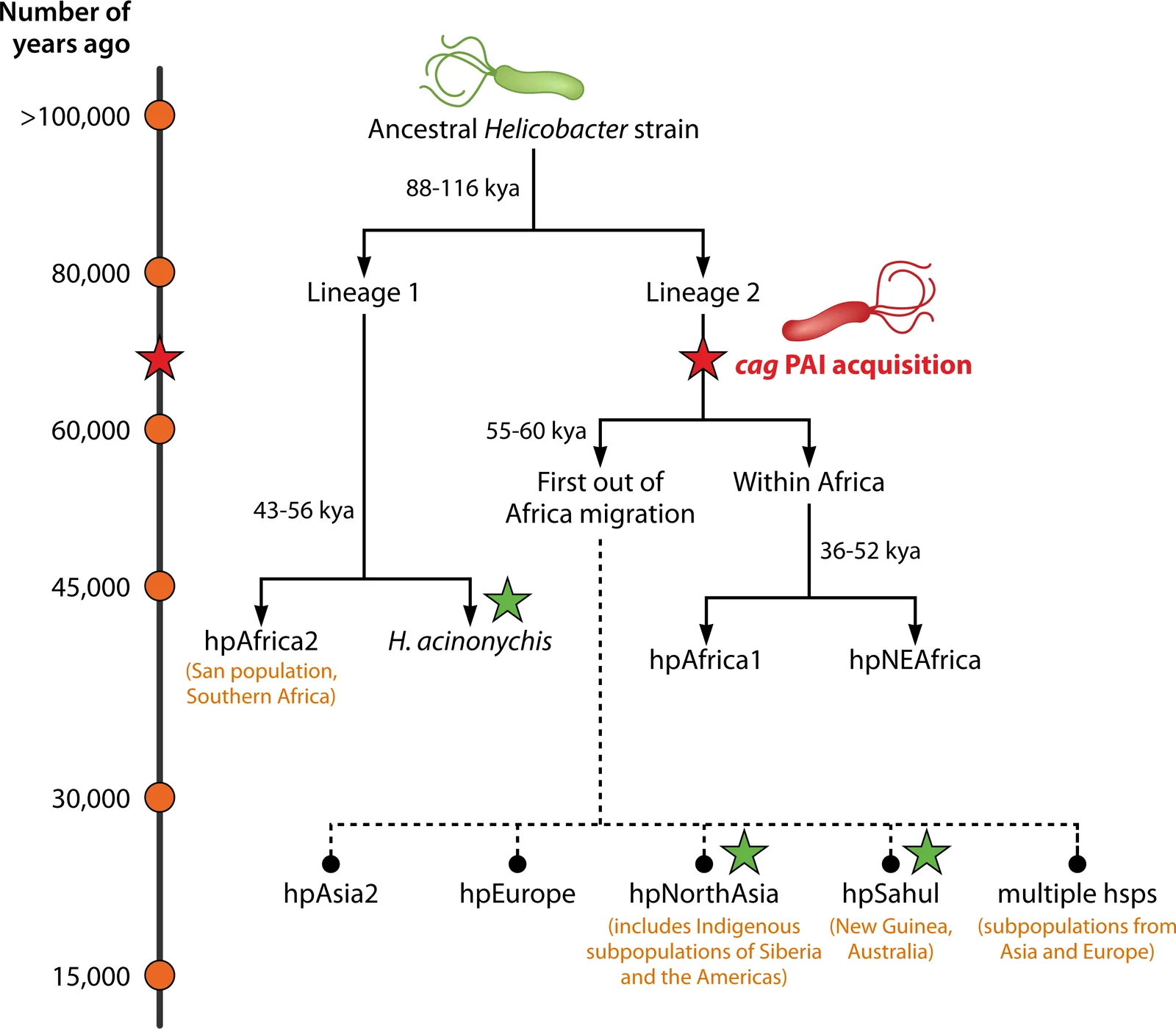
Timeline showing major steps in *H. pylori* evolution. Time points for key events were estimated based on MLST analyses of *H. pylori* genetic variation (75). The figure shows geographically distinct *H. pylori* populations (hp) or subpopulations (hsps) that colonize humans, as well as *H. acinonychis*, which colonizes large cats. Green stars indicate *H. pylori* populations that exhibit features characteristic of the Hardy ecospecies (77). The exact timepoints for the divergence of hpAsia2, hpEurope, hpNorthAsia, hpSahul, and other subpopulations are not known (represented by dotted lines) but are estimated to have occurred within the past 50 kya (75). Created with BioRender.com.

A current model proposes that humans have been colonized with *H. pylori* for more than 100,000 years (75–77). An analysis of *H. pylori* strains isolated in Africa suggested that an ancestral African *Helicobacter* population split into two main lineages about 100,000 years ago (Fig. 2) (75). One lineage yielded the group of *H. pylori* strains classified as hpAfrica2, which currently colonizes the San human population in Southern Africa. Modern human populations began migrating out of Africa in several waves, beginning about 60,000 years ago, and *H. pylori* strains from the second lineage accompanied their human hosts during these migrations (78, 79). The early out-of-Africa migrations represented bottleneck events due to low *H. pylori* population sizes (79). *H. pylori* strains have subsequently evolved within human population groups throughout the world, resulting in geographic variations that are currently detectable among *H. pylori* strains (75, 78, 80).

Genetic variations among *H. pylori* strains have been used to trace human migrations in many different parts of the world (79, 81–87). As one example, genetic variations in housekeeping genes of *H. pylori* strains isolated in East Asia, Australia, and South Pacific islands have been analyzed to trace the migrations of humans throughout the Pacific (83). These studies revealed two distinct groups of *H. pylori* strains (hpSahul and hspMaori), corresponding to prehistoric migrations to New Guinea and Australia (hpSahul) and to the Polynesian islands (hspMaori) (83). As another example, comparative genetic studies of *H. pylori* strains from Siberia and Indigenous American populations provided evidence that the entry of these *H. pylori* strains into the Americas was linked to a single migration event (87).

### *H. pylori* geographic populations

*H. pylori* strains isolated from human stomachs in different parts of the world exhibit striking differences in genetic characteristics. Early studies of *H. pylori* geographic diversity analyzed strains using multilocus sequence typing (MLST) of seven housekeeping genes: *atpA*, *efp*, *mutY*, *ppa*, *trpC*, *ureI*, and *yphC* (80, 88). The housekeeping genes used for MLST analysis are present in all *H. pylori* strains and are not predicted to be subject to positive selective forces. These early MLST studies led to the identification of *H. pylori* population groups localized in Europe, East Asia, or Africa, as well as numerous subpopulations (80). Subsequent MLST studies refined the designation of *H. pylori* population groups (78, 83). More recently, whole-genome sequence analyses of more than a thousand *H. pylori* strains, isolated in more than 50 countries, have been employed to provide an in-depth analysis of *H. pylori* geographic diversity (46, 77, 89, 90). One study classified the whole-genome sequences into four main population clusters (Southwest Europe, Northern and Central Europe, Western and Southern Africa, and Asia) and 17 subpopulations (46). Another study designated seven main population groups (hpAfrica1, hpAfrica2, hpAsia2, hpEurope, hpNEAfrica, npNorthAsia, and hpSahul), along with several subpopulations (77). The populations known as hpAfrica2 (found in Southern Africa) and hpSahul (found in New Guinea and Australia) are highly differentiated compared to the other populations (46, 83). Whole-genome sequence analyses also led to the identification of a distinct group of *H. pylori* strains known as the “Hardy ecospecies,” which likely arose more than 100,000 years ago (77). Strains belonging to the Hardy ecospecies have mainly been isolated from Indigenous populations (77). These strains are characterized by fixed SNPs in about 100 genes, genes encoding two urease enzymes (using either nickel or iron as a cofactor), and three copies of *vacA* (encoding secreted proteins) (77).

### Impact of *H. pylori* geographic variation on gastric cancer risk

Analyzing the potential role of geographic variations among *H. pylori* strains as a determinant of gastric cancer risk represents a substantial challenge. Thousands of *H. pylori* genetic polymorphisms and many different geographic regions could potentially be investigated. Thus far, investigations have focused mainly on geographic variations in *H. pylori* genes known to encode proteins that have roles in *H. pylori*–host interactions (68, 69). Since the incidence of gastric cancer is very high in parts of East Asia (14, 17), many studies have compared East Asian strains (especially from Japan or Korea) with Western strains (especially from the United States and Western Europe). These comparisons are facilitated by the relatively high level of genetic similarity that exists among East Asian strains, which has been attributed to relatively little admixture of East Asian strains with non-Asian strain populations subsequent to early human migrations out of Africa (79). There have been relatively few experimental studies conducted with strains from other parts of the world, and the relationship between the Hardy ecospecies and gastric cancer has not been investigated. The paucity of studies analyzing genetic features and phenotypic properties of *H. pylori* strains from diverse geographic locations limits our understanding of how geographic differences among *H. pylori* strains impact gastric cancer risk. In the following sections, we discuss *H. pylori* geographic variations linked to gastric cancer risk, focusing on genetic variations that are known to have functional consequences relevant to *H. pylori*-host interactions, based on experimental studies.

## *H. PYLORI* GEOGRAPHIC VARIATIONS RELEVANT FOR GASTRIC CANCER PATHOGENESIS

### Prevalence of the *cag* pathogenicity island

The *cag* pathogenicity island (*cag* PAI) is a 40-kilobase chromosomal region present in some *H. pylori* strains but not others (91, 92). One gene in the *cag* PAI (*cagA*) encodes a secreted effector protein (CagA) (93, 94), and other *cag* PAI genes encode components of a type IV secretion system (Cag T4SS) that spans the inner and outer membranes of *H. pylori* (95–98). The Cag T4SS is required for secretion of CagA, as well as the delivery of non-proteinaceous substrates (lipopolysaccharide [LPS] metabolites, peptidoglycan, and DNA), across the bacterial envelope into host cells (54, 92, 99). Delivery of CagA into gastric epithelial cells leads to disruption of multiple cell signaling pathways (100–104). Entry of LPS metabolites such as ADP-heptose into host cells triggers activation of the ALPK1-TIFA signaling pathway, leading to activation of NF-kB and production of proinflammatory cytokines such as interleukin (IL)-8 (105–109). Entry of peptidoglycan into host cells leads to activation of Nod1 (110, 111). Delivery of *H. pylori* DNA into host cells triggers activation of Toll-like receptor 9 (TLR9) (112–114).

The G + C content of the *cag* PAI is substantially lower than the G + C content of the rest of the *H. pylori* genome (115, 116), which suggests that the *cag* PAI was acquired through horizontal gene transfer. The *cag* PAI was likely acquired by *H. pylori* only once, from an unknown source (47, 91), resulting in insertion of the exogenous DNA into the *H. pylori* glutamate racemase locus. The *cag* PAI has not been detected in *H. pylori* strains classified as hpAfrica2 (47, 91, 117), and it has not been detected in other *Helicobacter* species (118). Therefore, the acquisition of the *cag* PAI by an ancestral *H. pylori* strain probably occurred after the differentiation of ancestral *H. pylori* into hpAfrica2 and non-hpAfrica2 lineages (about 100,000 years ago) and prior to the migration of modern humans out of East Africa about 60,000 years ago (Fig. 2) (75, 91).

The overall order and arrangement of genes within the *cag* PAI are relatively well conserved among *H. pylori* strains, but genetic rearrangement (sometimes mediated by insertion of transposable elements) or loss of genes within the *cag* PAI has been detected in some strains (47, 91, 115, 119). The primary functions of the *cag* PAI (secretion of CagA and non-protein substrates) are conserved in *H. pylori* strains that have intact *cag* PAIs, but there can be variations in the efficiency of these functions among strains (91).

Many studies have used *cagA* as a marker for the presence of the *cag* PAI instead of analyzing the entire PAI. The presence of *cagA* has been assessed by direct analysis of *H. pylori* isolates as well as by serologic assays to detect serum anti-CagA antibodies. Because individuals can be simultaneously colonized with both *cagA*-positive and *cagA*-negative strains (120), the analysis of single-colony isolates can potentially fail to detect gastric colonization with *cagA*-positive strains, and serologic testing represents a more sensitive detection method. Using multiple methodological approaches, epidemiologic studies have shown that gastric colonization with *cag* PAI-positive *H. pylori* strains is associated with an increased risk of gastric cancer or peptic ulcer disease, compared to colonization with *cag* PAI-negative strains (22, 70, 92, 121–124). Consistent with the results of epidemiologic studies, experiments using a Mongolian gerbil model have shown that CagA and the Cag T4SS have key roles in the pathogenesis of gastric cancer and gastric ulceration. Specifically, Mongolian gerbils experimentally infected with a wild-type CagA-producing strain commonly develop severe gastric inflammation and gastric cancer; *cagA* mutant strains or mutants with defects in Cag T4SS activity are able to colonize the gerbil stomach, but they do not cause gastric cancer (27, 28, 30–33).

**Fig 3:**
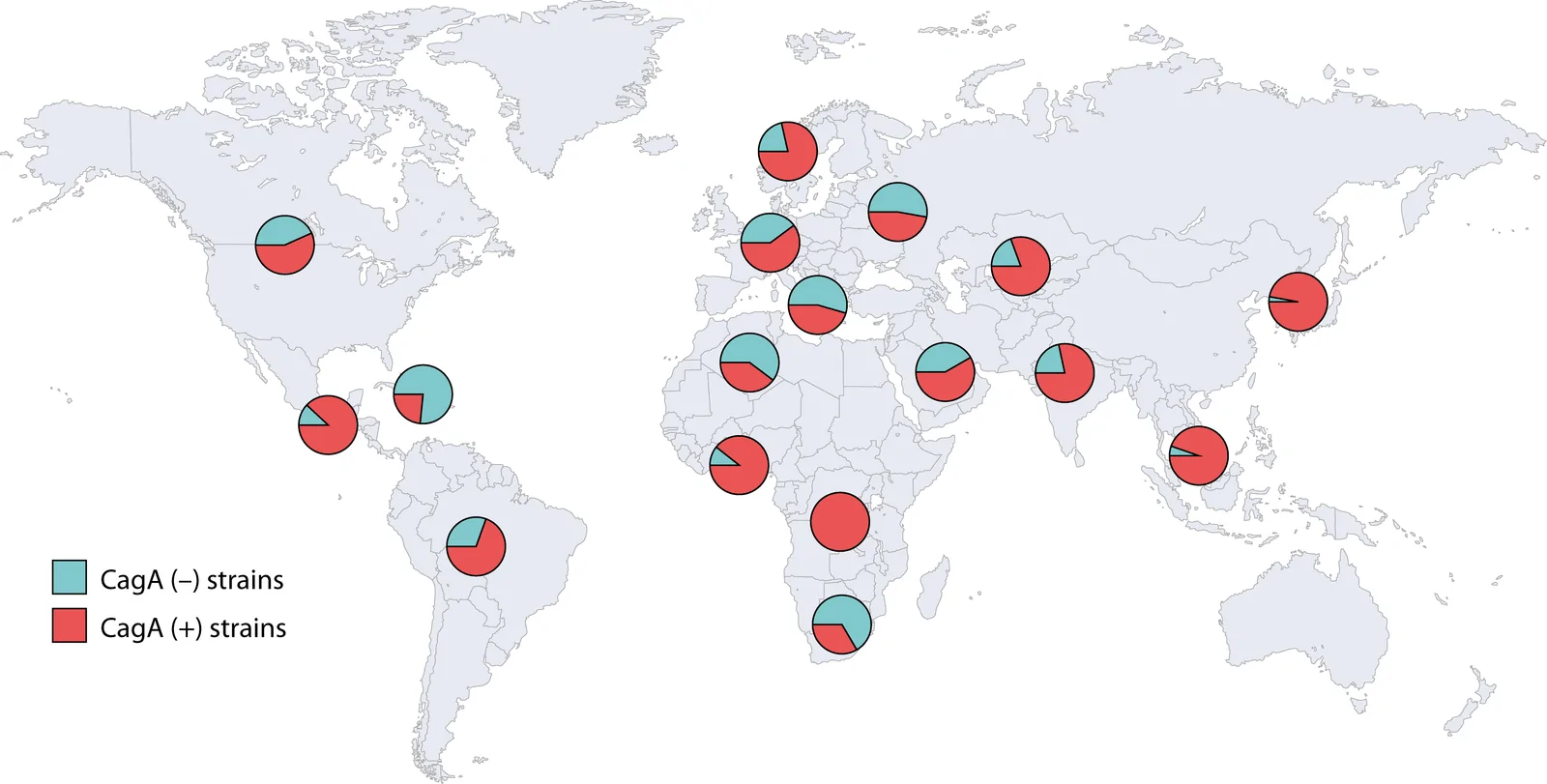
Geographic variations in *H. pylori cagA* status. *H. pylori* strains were classified as *cagA*-positive or *cagA*-negative, based on genome sequence analysis (46). The numbers of analyzed *H. pylori* genomes from Africa and Australia were substantially lower than the numbers from other geographic regions. Created with BioRender.com.

The prevalence of the *cag* PAI varies in *H. pylori* strains from different parts of the world. About 50%–80% of Western *H. pylori* isolates (from the Americas and Europe) contain the *cag* PAI*,* and the remainder lack the *cag* PAI (46, 91, 123, 125–127). In contrast, most East Asian isolates harbor an intact *cag* PAI (46, 91, 126–130). Therefore, the proportion of *cagA*-positive *H. pylori* strains is substantially higher in parts of East Asia (which have high rates of gastric cancer) than in many Western countries (Fig. 3).

The nucleotide sequences of genes within the *cag* PAI exhibit variation among *H. pylori* strains, and as expected, geographic variations are detectable (91, 131). Among genes within the *cag* PAI, *cagA* exhibits the highest level of sequence variation (for example, the highest Ka/Ks value) (91, 132). One analysis reported that 13 genes in the *cag* PAI have evolved under diversifying selection, based on the use of multiple criteria; *cagA*, *cagY*, and *cagQ* had the highest percentages of codons exhibiting a high likelihood of positive selection (91). In another study, polymorphisms in multiple *cag* PAI genes exhibited evidence of fixation within specific human populations: *cagF* (a putative chaperone for CagA) had the highest fixation index (90). Several genes encoding structural components of the Cag T4SS (CagM, CagV, and CagH) exhibited evidence of fixation in the latter study but did not show evidence of diversifying selection in the former study; *cagX, cagL, cagN,* and *cag4* were identified in both studies. The functional consequences of specific sequence variations in *cagA* have been extensively investigated *in vitro* and have been linked to gastric disease risk, as described in subsequent sections (90, 91). Polymorphisms in several other *cag* PAI genes (including *cagL, cagC, cag3,* cagI, and *cagβ*) have also been correlated with gastric cancer risk (133–135), but thus far, there has been relatively little investigation of functional consequences of variations in the sequences of *cag* PAI genes, other than *cagA*.

### CagA

#### Overview of CagA actions linked to gastric cancer pathogenesis

CagA, an effector protein encoded by the *cag* PAI, is secreted by the Cag T4SS and enters host cells (100–104, 136, 137). Five distinct CagA domains have been described (93, 94). Domains I–III, comprising the N-terminal portion of the protein and accounting for about 70% of the CagA structure, have been resolved by crystallography (93, 94). Domains IV and V are localized in the intrinsically disordered C-terminal region of CagA (93). Variations in the molecular mass of CagA (ranging from 130 to 145 kDa) are mainly attributed to differences in the C-terminal regions.

**Fig 4:**
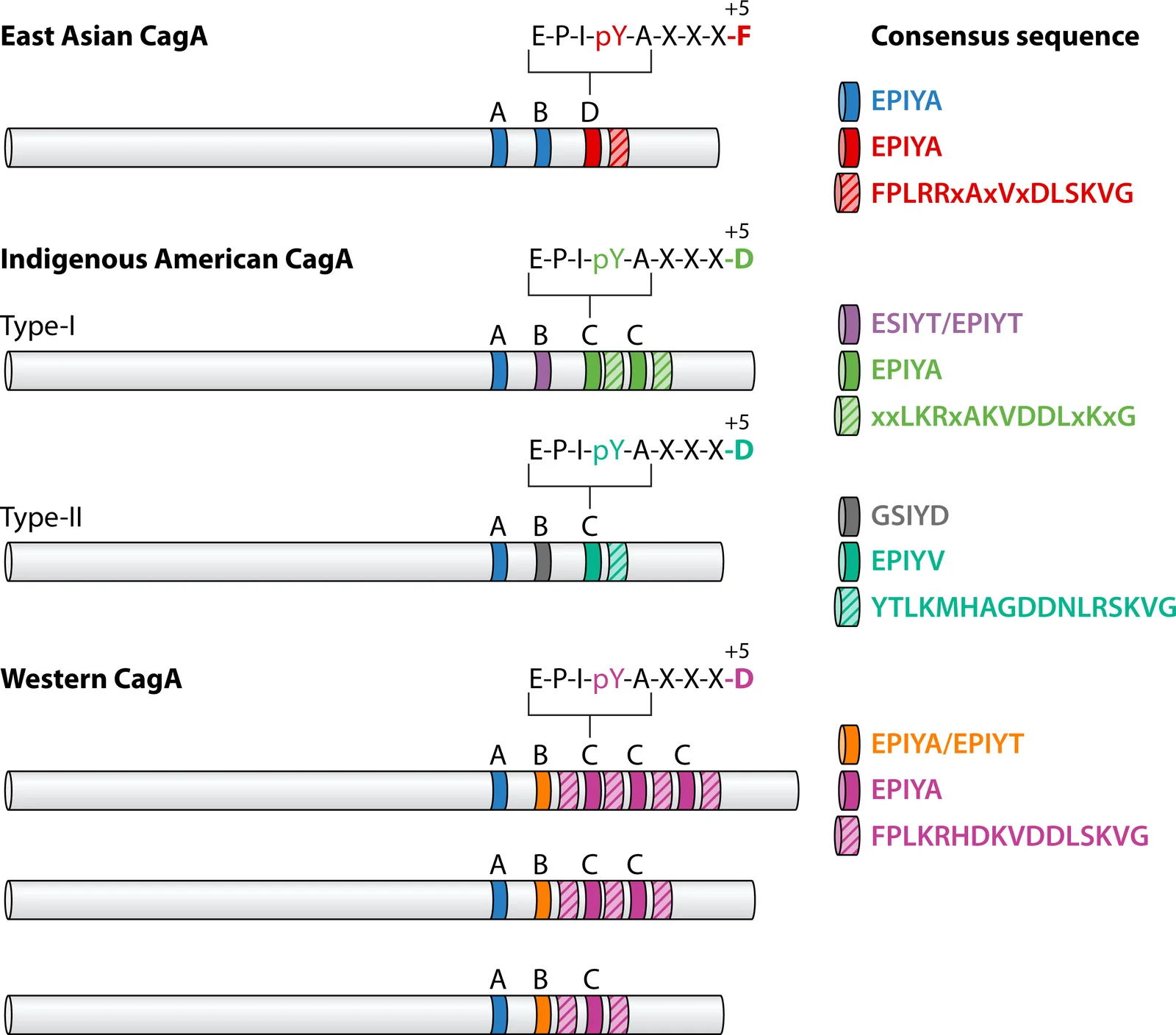
Variations in *H. pylori* CagA tyrosine phosphorylation and multimerization motifs. Four types of CagA tyrosine phosphorylation motifs (solid colored boxes) are recognized (designated EPIYA-A, EPIYA-B, EPIYA-C, and EPIYA-D). Colored pY letters indicate sites of tyrosine phosphorylation that influence CagA binding to SHP-2. CagA multimerization motifs (striped boxes) are localized adjacent to EPIYA-C or EPIYA-D motifs (146). EPIYA-D motifs are characterized by a unique phenylalanine (F) residue at the +5 position from the tyrosine. Western and J-Western CagA proteins typically contain EPIYA-A, -B, and -C motifs (with varying numbers of EPIYA-C motifs). Indigenous American CagA (type-I and type-II) contain EPIYA-A, -B, -C motifs (with modified EPIYA-B motifs and varying numbers of EPIYA-C motifs), and East Asian CagA proteins (from Japan, Korea, and parts of China) are characterized by EPIYA-A, -B, and -D motifs. Consensus sequences are shown. Created with BioRender.com.

Upon entry into human gastric epithelial cells, CagA can undergo tyrosine phosphorylation by Src and Abl kinases at specific Glu-Pro-Ile-Tyr-Ala (EPIYA) sequence motifs (100–104, 136, 138), localized within Domain IV of CagA (Fig. 4). Within host cells, CagA also undergoes multimerization, which is mediated by CagA multimerization (CM) motifs localized within Domain IV of CagA (Fig. 4) (139–143). Both the phosphorylated and unphosphorylated forms of CagA can interact with specific intracellular host cell proteins, leading to disruptions in cell signaling pathways (100–104, 137). CagA is reported to interact with at least 20 different host cell proteins (103, 104, 144). The interaction of phosphorylated CagA with the tyrosine phosphatase SHP-2 has been extensively studied (145). This interaction leads to the activation and deregulation of downstream signaling pathways, accompanied by changes in cell morphology (termed the “hummingbird phenotype”) and increased cell motility (100–102, 104).

Epidemiologic studies have shown that gastric colonization with *cagA*-positive *H. pylori* strains is associated with an increased risk of gastric cancer, premalignant gastric lesions, and peptic ulcer disease, compared to colonization with *cagA*-negative strains (22, 92, 121–124). Among *cagA*-positive strains, those with high levels of *cagA* expression are associated with an increased risk of premalignant lesions compared with those with lower levels of *cagA* expression (147, 148). CagA has a key role in the pathogenesis of *H. pylori*-induced gastric cancer in the Mongolian gerbil model (27, 28, 30–33), and transgenic mice expressing CagA develop gastric tumors and hematopoietic neoplasms (149). Therefore, CagA has been designated as a bacterial oncoprotein (100, 102, 150).

#### *cagA* genetic diversity

Phylogenetic analyses of *cagA* genes from *H. pylori* strains isolated in different parts of the world have revealed several main clusters of sequences, including East Asian *cagA* sequences (typically found in strains classified as hspEastAsia, based on analysis of housekeeping gene sequences), Western *cagA* sequences (commonly found in Europe, Africa, and the Americas), Indigenous American forms of *cagA* (two types—type I and type II), and “J-Western” forms of *cagA* (found commonly in Okinawa, Japan) (91, 146, 151–155). These lineages of CagA proteins exhibit distinguishable differences in activity in cell culture assays, as described in subsequent sections. Most studies have focused on differences in activity between East Asian forms of CagA and Western forms of CagA. There has been relatively little study of the activity of Indigenous American forms of CagA (146), and thus far, there have not been any investigations of the activity of J-Western forms of CagA. East Asian CagA sequences are the predominant type in regions of East Asia that have a very high incidence of gastric cancer (e.g., Korea and Japan). The presence of J-Western forms of CagA in Okinawa has been associated with a lower incidence of gastric cancer in Okinawa than in other parts of Japan (154, 156).

Among genes within the *cag* PAI, *cagA* exhibits the highest level of sequence variation (91, 132). Some of the most striking geographic variations in *cagA* occur in regions encoding tyrosine phosphorylation (EPIYA) motifs and regions encoding CagA multimerization motifs (Fig. 4) (100, 102, 150). Geographic differences in these motifs influence CagA activities (141, 146, 157–160) and have been linked to differences in gastric disease risk (124, 146, 154, 161–164). The functional consequences of most of the other *cagA* variations linked to gastric cancer risk and other positively selected variations in *cagA* (91, 134, 135) have not been investigated.

#### Variations in CagA phosphorylation motifs

Within host cells, CagA undergoes phosphorylation at conserved tyrosine residues within EPIYA sequence motifs (Glu-Pro-Ile-Tyr-Ala), which are localized in the unstructured C-terminal region of CagA. CagA proteins from different *H. pylori* strains vary in the type and number of EPIYA phosphorylation motifs (102, 152). These motifs have been classified as EPIYA-A, EPIYA-B, EPIYA-C, or EPIYA-D, based on the adjacent sequences (Fig. 4). The characteristics of CagA EPIYA motifs (e.g., the number of EPIYA motifs and the flanking sequences) influence CagA activity (102, 157, 158, 165, 166). In addition to the most common form of tyrosine phosphorylation motif (EPIYA), CagA proteins can contain variant phosphorylation motif sequences, including EPIYT, ESIYT, EPIYV, and GSIYD (146, 167).

Geographic variations in CagA phosphorylation motifs are illustrated in Fig. 4. The EPIYA-A motif is found in CagA proteins from *H. pylori* strains throughout the world, and the amino acid sequences of EPIYA-A motifs are highly conserved among *cagA*-positive *H. pylori* isolates (146). In contrast, the amino acid sequences of EPIYA-B motifs vary among *H. pylori* strains. EPIYA-B sequence variations include EPIYT (in CagA from Western and type-I Indigenous American strains), ESIYT (in type-I Indigenous American strains), and GSIYD (in type-II Indigenous American strains) (146, 167). EPIYA-C and EPIYA-D motifs are localized adjacent to CagA multimerization sequence motifs (discussed in the next section). EPIYA-C motifs are found in CagA proteins from Western strains, J-Western strains, and Indigenous American *H. pylori* isolates, and can be present in multiple copies (Fig. 4). The EPIYA-C motif has a characteristic aspartic acid (D) residue at the +5 position relative to the tyrosine (146, 153). EPIYA-D motifs are found almost exclusively in East Asian CagA proteins (102, 157, 158, 166) and are characterized by a unique phenylalanine (F) residue at the +5 position. In summary, Western and J-Western CagA proteins typically display EPIYA-A, -B, and -C motifs (with varying numbers of EPIYA-C motifs), Indigenous American CagA display EPIYA-A, -B, -C motifs (with modified EPIYA-B motifs and varying numbers of EPIYA-C motifs), and East Asian CagA proteins contain EPIYA-A, -B, and -D motifs (Fig. 4).

EPIYA-C and EPIYA-D motifs in CagA are specifically phosphorylated by Src family kinases, while c-Abl kinases can phosphorylate all EPIYA motifs (138). A single CagA protein is phosphorylated at no more than two EPIYA motifs (138). In Western CagA, phosphorylation typically occurs on EPIYA-A and -C motifs, while East Asian CagA is preferentially phosphorylated on EPIYA-B and EPIYA-D motifs (100–102, 104).

Numerous studies have compared CagA EPIYA variants using biochemical or cell culture assays. East Asian CagA proteins demonstrate a stronger affinity for SHP-2 than Western CagA proteins. Specifically, the EPIYA-D motif of East Asian CagA binds SHP-2 more effectively than the EPIYA-C motif of Western-type CagA (157, 158, 161). The presence of a phenylalanine residue at the +5 position from the phosphorylated tyrosine residue in EPIYA-D of East Asian CagA (Fig. 4) increases its binding affinity for SHP-2 by as much as 100-fold (157, 158). The increased affinity of SHP-2 binding enhances CagA-induced effects on host cells, including changes in cell morphology (hummingbird phenotype) and cell invasion of the extracellular matrix (157, 158). The presence of multiple copies of the EPIYA-C motif in Western-type CagA can also enhance SHP-2 binding, resulting in enhanced CagA activity in cell culture assays (163, 168).

The presence of an alanine or a threonine residue at the +1 position relative to the tyrosine in EPIYA-B motifs can influence interactions with host cell phosphoinositol 3-kinase (PI3-kinase) and subsequent downstream signaling pathways (167). Specifically, CagA proteins containing EPIYT-B motifs have higher affinity for PI3-kinase than those containing EPIYA-B motifs, as the threonine side chain forms a hydrogen bond with asparagine at position 417 of PI3-kinase. CagA proteins containing an EPIYT-B motif stimulate increased phosphorylation of AKT compared to CagA proteins containing the EPIYA-B motif, along with attenuated induction of the hummingbird phenotype (167).

Variation in EPIYA tyrosine phosphorylation motifs has been linked to gastric cancer risk (146, 164). In regions of Asia where *H. pylori* strains contain either EPIYA-D or EPIYA-C CagA motifs, the strains with EPIYA-D motifs are associated with a higher risk of gastric cancer (154, 161, 162). Among Western *H. pylori* strains, those producing CagA proteins with higher numbers of EPIYA-C motifs have been associated with an increased risk of gastric premalignant lesions and gastric cancer (124, 163). Among Western strains, CagA proteins containing EPIYA-B tyrosine phosphorylation motifs are associated with an increased risk of gastric cancer compared to CagA proteins containing EPIYT-B tyrosine phosphorylation motifs (167). These epidemiologic observations correlate with the observed differences in activity of CagA variants in biochemical assays.

#### Variations in CagA multimerization motifs

The C-terminal portion of CagA also contains conserved CM motifs, localized in association with EPIYA-C motifs and EPIYA-D motifs (139–143) (Fig. 4). The CM motif, consisting of about 16 amino acids, is required for the formation of CagA dimers at the host cell plasma membrane. The CM motif influences the affinity of CagA for several host cell proteins, including SHP-2 and PAR1b (a protein that regulates tight junctions and cell polarity) (139). Therefore, this region of CagA has also been designated as a “conserved repeats responsible for phosphorylation-independent activity” (CRPIA) motif, required for interactions between non-phosphorylated CagA and host cell proteins (169).

*H. pylori* strains from different geographic regions exhibit variability in the number and sequences of CagA CM motifs (Fig. 4). East Asian CagA proteins contain only one CM motif, positioned distal to the EPIYA-D motif (140). The number of CM motifs present in Indigenous American CagA proteins correlates with the number of EPIYA-C repeats; CM motifs in these strains are localized distal to each EPIYA-C motif. Western-type CagA proteins have an additional CM motif at the N-terminus of the first EPIYA-C, along with CM motifs distal to each EPIYA-C (Fig. 4). The consensus sequence for the CM motif of East Asian CagA (FPLRRxAxVxDLSKVG) is related to that of Western-type CagA (FPLKRHDKVDDLSKVG), but CagA proteins containing an East Asian CM motif exhibit a twofold stronger binding affinity for PAR1b, resulting in stronger alterations to cell polarity, compared to CagA proteins containing a Western CM motif (141). Among Western CagA proteins, PAR1b binding is directly proportional to the number of CM sequences (141). The CM motif of type-II (YTLKMHAGDDNLRSKVG) Indigenous American CagA is distinct from that of type-I (xxLKRxAKVDDLxKxG), East Asian, and Western CM motifs (Fig. 4). CM motifs of type-II Indigenous American strains exhibit impaired binding to PAR1b (141, 146, 160) and reduced ability to induce IL-8 and MUC2 mucin expression in gastric epithelial cells (146). Therefore, the distinct features of the CM motif in type-II Indigenous American CagA have been linked to attenuated activity of CagA in these strains (146) (Fig. 4). In summary, numerous studies have shown that geographic variations in both CagA phosphorylation motifs and CM motifs influence the intracellular actions of CagA.

#### Variation in *cagA* copy numbers

Most *cagA*-positive *H. pylori* strains contain a single copy of *cagA*, but some strains possess multiple copies (up to four copies per strain), resulting in increased levels of CagA production (170, 171). The levels of CagA production can also be influenced by genetic variation in sequences upstream of the CagA translational start site (147, 148). *H. pylori* strains encoding multiple *cagA* genes have been identified in East Asian and a subset of Western strains (171). Amplification of the *cagA* gene typically occurs through homologous recombination events that duplicate a genetic region flanking the *cagA* gene, termed the “*cagA* homologous area—upstream/downstream,” or (CHA)-ud (171). The number and arrangement of CHA-ud sequences are variable among *H. pylori* strains. Increased *cagA* copy numbers have been correlated with increased consequences of CagA activity in AGS gastric epithelial cell co-culture assays (cell elongation and IL-8 secretion) (170). Thus far, it is not known if *cagA* copy number influences the risk of gastric cancer.

### VacA

VacA is a pore-forming exotoxin secreted by *H. pylori* (172–174). The most prominent effect of VacA on cultured eukaryotic cells is an expansion of late endosomal compartments, termed vacuolation. VacA can also cause changes in plasma membrane permeability, mitochondrial dysfunction, and apoptosis or necrosis in certain cell lines (172–174). In addition to its effects on gastric foveolar epithelial cells (surface mucous cells), VacA can cause alterations in several types of immune cells (including T cells, dendritic cells, and mast cells) and parietal cells (172–174).

**Fig 5:**
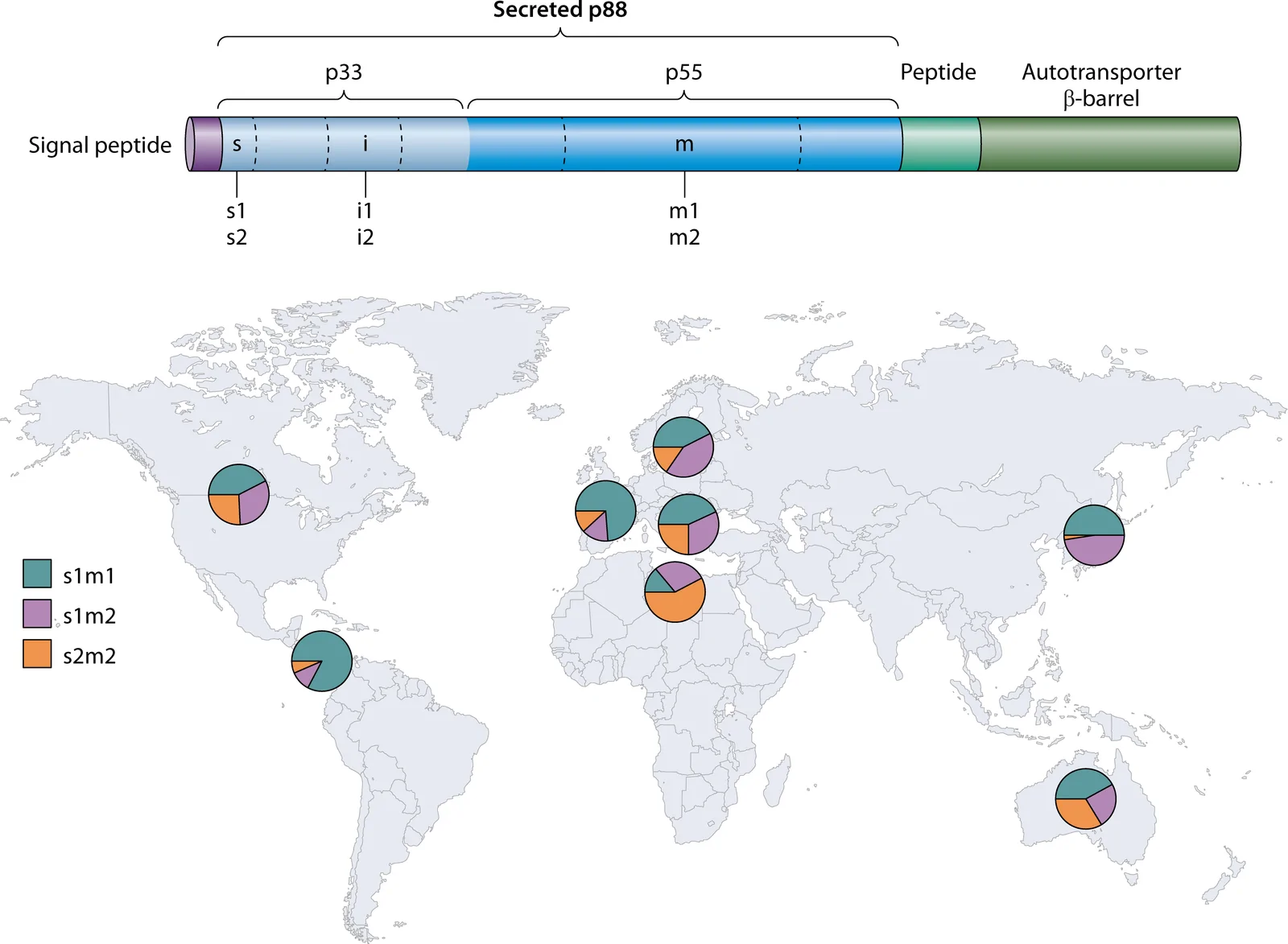
Global variations in *H. pylori vacA* genotypes. The top panel shows a schematic diagram of the VacA protein. A 140 kDa precursor protein undergoes proteolytic cleavage to yield the secreted 88 kDa VacA protein. Regions exhibiting high levels of sequence diversity are indicated (s, i, and m regions). The bottom panel shows classification of *vacA* genes in different regions of the world as s1m1, s1m2, or s2m2, based on PCR analyses (126). Created with BioRender.com.

The *vacA* gene encodes a 140 kDa protein that undergoes proteolytic cleavage to yield a C-terminal autotransporter β-barrel that inserts into the *H. pylori* outer membrane and an 88 kDa toxin secreted into the extracellular space (Fig. 5) (172–175). The secreted 88 kDa VacA protein consists of two domains: an N-terminal p33 domain and a C-terminal p55 domain. The secreted 88 kDa VacA protein can assemble into oligomeric structures (176, 177), and it inserts into host cell membranes to form anion-selective membrane channels (178–180).

All *H. pylori* strains contain a chromosomal *vacA* gene, but there is a high level of sequence diversity among *vacA* alleles (90, 181–183) and *vacA* alleles exhibit signatures of strong positive selection (90, 181–183). Sequence variations in highly polymorphic regions influence toxin activity (184–186). The three main regions of sequence diversity are the signal sequence region (s-region), the intermediate region (i-region), and the middle region (m-region) (Fig. 5). The s-region extends from the signal sequence into the amino-acid terminus of the secreted 88 kDa toxin. Two main genetic variants of the s-region have been reported: s1 and s2. The s1 and s2 forms of VacA undergo signal sequence processing at different sites; s2 forms contain a hydrophilic 12 amino acid extension at the N-terminal end of the 88 kDa secreted protein, which is absent from s1 forms (184–186). The m-region is a large segment (about 280 amino acids) within the p55 domain. There are two main families of m-region alleles: m1 and m2 (184). Varying combinations of the s and m *vacA* allelic types are commonly identified (for example, s1m1, s1m2, and s2m2), but s2m1 forms are extremely rare (126) (Fig. 5). An additional region of diversity, termed the intermediate region (i-region), is located within the portion of *vacA* encoding the p33 domain (187). There are two main types of *vacA* i-region alleles: i1 and i2 (187).

Type s1 forms of VacA are highly active in cell culture assays for vacuolation and are transcribed at higher levels than s2 forms (184–186, 188). Type s2 forms of VacA lack detectable vacuolating activity in cell culture assays using HeLa, Vero, AGS, and RK13 cells and have an attenuated capacity to form membrane channels (184–186). Both m1 and m2 forms of VacA have detectable vacuolating activity, but m1 forms of VacA have increased vacuolating activity compared to m2 forms in HeLa cells (189–192). The observed difference in activity has been attributed to lower levels of m2 VacA binding to cells compared to the binding of m1 forms (189, 192). One study reported that type i1 forms of VacA have stronger vacuolating activity than i2 forms in RK-13 cells (187). Type i1 forms of VacA also have increased activity compared to type i2 forms in Jurkat T-cells (193).

Numerous epidemiologic studies have reported an association between type s1, i1, or m1 forms of *vacA* and gastric cancer or peptic ulcer disease (22, 124, 184, 187, 194, 195). Several additional regions of diversity within the *vacA* gene have been reported to correlate with gastric histologic alterations or disease status (196), but the functional consequences of variations in these other regions have not been investigated. The contributions of different forms of VacA to *H. pylori* gastric disease in animal models remain incompletely understood, but in one study, strains producing s1i1 forms of VacA induced greater levels of gastric inflammation and metaplasia in a mouse model than s2i2 forms of VacA (195).

Similar to *cagA* allelic variants*, vacA* genotypes vary geographically (126, 197, 198). Early phylogenetic analyses revealed three main clusters of *vacA* sequences (181, 199). More recent analyses of larger numbers of strains have identified additional clusters of *vacA* sequences, some of which correspond to geographically distinct clusters of alleles (90). The three main clusters were defined as Group 1: non-Asian strains (origins in Australia, Kenya, the United States, and Europe), Group 2: Asian strains (origins in China and Japan), and Group 3: strains with a worldwide distribution. The *vacA* sequences in Groups 1 and 2 correspond to the s1m1 type, and the sequences in Group 3 correspond to m2 types. The phylogenetic separation of these *vacA* sequences correlates with sequence variations in a surface-exposed region of the VacA p55 domain, which is involved in binding to host cells. The sequence divergence in this region of *vacA* is markedly different from the diversity that exists among housekeeping gene sequences, and the divergent *vacA* sequences exhibit features consistent with strong positive selection (181, 183).

*H. pylori* strains found in regions of the world with high rates of gastric cancer tend to contain *vacA* alleles encoding the s1m1 form of VacA (126, 129, 200). Strains from East Asian countries almost exclusively carry s1 *vacA* alleles, and the m1 alleles are dominant compared to m2 (Fig. 5) (126, 197, 198, 200, 201). *H. pylori* strains isolated in France and Italy, North America, and Australia contain a higher proportion of s2 *vacA* alleles than strains from Asian countries (approximately 30% compared to <5%), and m2 *vacA* alleles are more common than m1 *vacA* alleles in the former regions (126).

### *H. pylori* outer membrane proteins

The *H. pylori* genome contains more than 50 genes that are predicted to encode outer membrane proteins (OMPs) (202). Several of these have key roles in *H. pylori*–host cell interactions. For example, BabA, SabA, HopQ, AlpA, and AlpB are adhesins that interact with receptors on host cells. The genes encoding most of these adhesins are variably present or absent among *H. pylori* strains (Table 1), and if present, there is variation among strains in the corresponding nucleotide sequences.

**TABLE 1:** Strain-specific *H. pylori* genetic features that correlate with the presence of the *cag* PAI

Gene	Description of product	Characteristics of the indicated gene in *H. pylori* strains that are:	
		*cag* PAI positive	*cag* PAI negative
*cagA*	Effector protein	Present	Absent
*vacA*	Secreted toxin	s1 forms	s2 forms
*babA*	OMP	Present	Absent
*homB*	OMP	Present	Absent
*hopH* (*oipA*)	OMP	In-frame	Out-of-frame
*hopQ*	OMP	Type I form	Type II form

BabA is an OMP adhesin that binds to Lewis b and related blood group receptors on host cells, as well as MUC5AC mucin (203–205). BabA-mediated adherence to host cells augments Cag T4SS-dependent signaling (206). Experiments in transgenic mice expressing Lewis b showed that *H. pylori* adherence to this receptor can influence levels of gastric inflammation and parietal cell loss (207). The *babA* gene can be either present or absent in *H. pylori* strains, and if present, there can be variation in the number of copies (208). There is also variation among *H. pylori* strains in *babA* sequences (209). Variation in *babA* sequences influences the affinity of BabA binding to ABO blood group antigens (209, 210). Several studies have reported that gastric cancer occurs more commonly in individuals colonized with *babA*-positive *H. pylori* strains than among those colonized with *babA*-negative strains (70, 211–213).

BabA-positive *H. pylori* strains are more common in East Asian countries than in Western countries (198, 214–217). There are also geographic differences in the binding properties of BabA. The BabA proteins produced by most *H. pylori* strains bind multiple fucosylated blood group antigens (A, B, and O) and are classified as “generalists.” In contrast, the BabA proteins produced by other *H. pylori* strains are specialized for binding blood group O antigens and are classified as “specialists” (209). “Specialist” forms of BabA are commonly present in Indigenous American strains of *H. pylori*, which coincides with a predominance of blood group O in Indigenous American human populations. Therefore, the blood group-binding properties of BabA in *H. pylori* strains correlate with the dominant blood group antigens of the corresponding human hosts. Interestingly, the *babA* sequences in *H. pylori* strains isolated from Latin American subpopulations exhibit unexpectedly high relatedness to *babA* sequences from hspEastAsia strains (90). At present, it is not known if this relationship reflects convergent evolution or horizontal transfer events.

HopQ is an OMP adhesin that binds to CEACAM receptors on host cells (218, 219) and contributes to Cag T4SS activity (218–222). HopQ binding to CEACAM receptors disrupts CEACAM dimerization, which may impact CEACAM-mediated cell adherence and signaling (223, 224). Similar to *babA*, *hopQ* genes can be present or absent in *H. pylori* strains, and there can be variation in the number of copies of *hopQ*. There is also substantial variation in *hopQ* sequences. Two main families of *hopQ* alleles have been described, designated type I and type II (225). Type I and type II *hopQ* alleles are 75%–80% identical in nucleotide sequences and encode outer membrane proteins that are <75% identical in amino acid sequences (225). Type I HopQ proteins can interact with multiple human CEACAM receptors, including CEACAM1, CEACAM3, CEACAM5, and CEACAM6 (219). Type I HopQ proteins from some strains do not bind CEACAM6 (223). Type II HopQ proteins bind with high affinity to CEACAM1, but not to CEACAM6 (223). In comparison to type I HopQ, type II HopQ has a sixfold higher binding affinity to CEACAM1 (223, 226).

*H. pylori* strains containing type I *hopQ* alleles have been associated with an increased risk of gastric premalignant alterations, gastric cancer, or peptic ulcer disease, compared to strains containing type II *hopQ* alleles or strains lacking *hopQ* (70, 225, 227, 228). Most East Asian *H. pylori* strains contain type I forms of *hopQ*, while Western strains contain either type I or type II *hopQ* alleles (229). The differential risk of severe gastric disease associated with type I and type II HopQ proteins might be attributable to differences in the CEACAM binding properties of these proteins.

HopH (also known as OipA) is an OMP that contributes to *H. pylori* adherence to gastric epithelial cells (230) and *H. pylori*-induced stimulation of IL-8 production by gastric epithelial cells (231, 232). The host cell receptor for HopH is not known. The *hopH* gene contains dinucleotide tracts and consequently undergoes phase variation (230–232). Therefore, *hopH* can be either in-frame or out of frame. Several studies have reported an association between *H. pylori* strains containing in-frame *hopH* genes and gastric cancer (212, 233, 234). In-frame *hopH* alleles are predominant in East Asian strains (217, 231, 235–237).

HomB is another outer membrane protein that has been linked to gastric cancer risk (198, 238, 239). In comparison to wild-type *H. pylori* strains, *homB* mutant strains exhibit reduced binding to human gastric epithelial cells and decreased ability to induce IL-8 secretion from these cells (240). The host cell receptor for HomB is not known. *H. pylori* strains containing *homB* have been linked to gastric cancer or peptic ulcer disease in Western populations (198, 240, 241), and a high proportion of East Asian *H. pylori* strains contain *homB* (198, 216, 217, 241–243).

*H. pylori alpA* and *alpB* are two closely related genes that are co-transcribed and present in nearly all *H. pylori* strains (232, 244). AlpA and AlpB contribute to *H. pylori* adherence to gastric epithelial cells and facilitate the binding of *H. pylori* to laminin, a prominent component of the extracellular matrix, including the basement membrane of cells (245, 246). The host cell receptor(s) for AlpA and AlpB are not known. These proteins also have a role in *H. pylori*-induced cytokine production by gastric epithelial cells (245). Recent studies indicate that these proteins mediate *H. pylori* agglutination, which might be relevant for biofilm formation (247). A comparative analysis of AlpA and AlpB amino acid sequences in East Asian and Western strains revealed ten amino acid positions in AlpA and five in AlpB that were unique to East Asian strains; the functional consequences of these polymorphisms have not been investigated (245). Phylogenetic analysis of *alpB* sequences in *H. pylori* strains isolated in the Americas revealed unexpectedly high levels of Asian ancestry in several Latin American subpopulations (86). In both East Asian and Western *H. pylori* strains, deletion of *alpA* and *alpB* diminishes the ability of the strains to stimulate IL-6 production (245). Interestingly, deletion of *alpAB* in East Asian strains but not Western strains resulted in reduced ability to stimulate IL-8 production in gastric epithelial cells (245). Similarly, *alpAB* in East Asian strains (but not Western strains) contributed to c-Jun activation (245).

In addition to variation among *H. pylori* strains in the OMP-encoding genes described above, there is also variation in genes encoding other OMPs (including *sabA* [212, 248]*, sabB, babB* [74], *homA, hopZ,* and *labA*), several of which function as adhesins. Relatively little is known about geographic variation in these OMP-encoding genes or their potential roles in gastric cancer pathogenesis.

### *H. pylori* lipopolysaccharide structure

**Fig 6:**
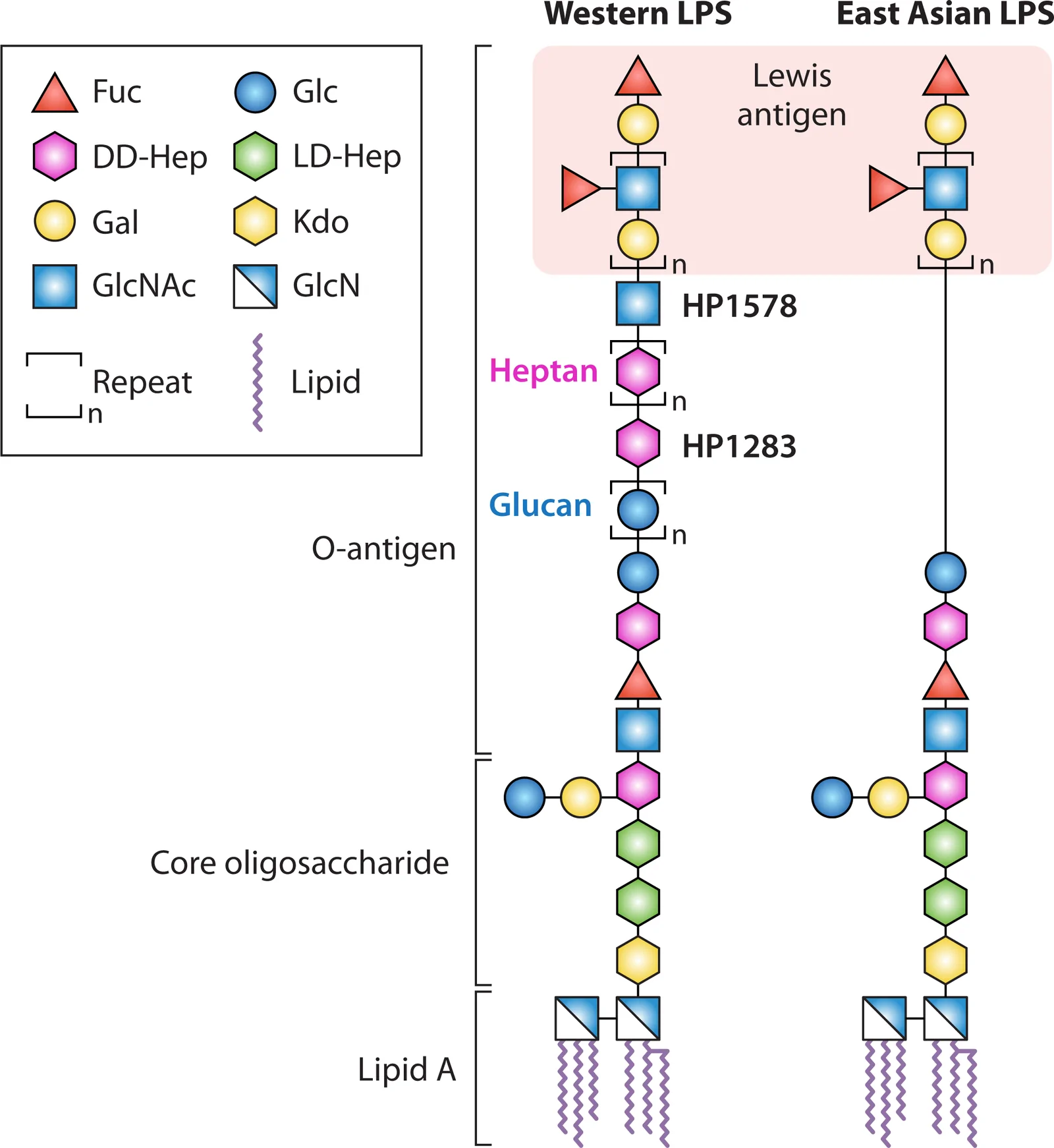
Variations in *H. pylori* lipopolysaccharide (LPS) structures. *H. pylori* strains classified as hpEurope commonly contain HP1578 and HP1283 genes, which encode glycosyltransferases. East Asian *H. pylori* strains typically lack the HP1578 and HP1283 genes and are unable to incorporate glucan and heptan polymers into the O-antigen structure (250). The figure shows cartoon depictions of the structures of these two different types of LPS. Three LPS domains are shown (lipid A, core oligosaccharide, and O-antigen).

Similar to lipopolysaccharide from other gram-negative bacterial species, *H. pylori* LPS is organized into three structural domains: lipid A, core oligosaccharide, and O-antigen (Fig. 6) (249–251). *H. pylori* LPS has specialized properties that facilitate *H. pylori* evasion of host innate immune defenses. In comparison to the lipid A of other gram-negative species, *H. pylori* LPS lipid A exhibits a different acylation pattern (tetra-acylated instead of hexa-acylated) and is dephosphorylated (251–254). These modifications diminish the affinity of *H. pylori* LPS for Toll-like receptor 4 (TLR4) and confer resistance to positively charged antimicrobial peptides (253, 255). The O-antigen of *H. pylori* LPS shares structural similarities with Lewis antigens found on host cells (256). Lewis antigen components of *H. pylori* LPS facilitate *H. pylori* binding to dendritic cell-specific intracellular adhesion molecule-3-grabbing non-integrin (DC-SIGN), a C-type lectin receptor on the surface of macrophages and dendritic cells (257). This interaction not only regulates dendritic cell function but also effectively suppresses pro-inflammatory responses, thereby contributing to the persistence of *H. pylori* within the host.

Geographic variations in *H. pylori* LPS structure and biosynthesis have been detected (250, 251, 258, 259). L-glycero-D-manno-heptose (LD-Hep) stereoisomeric units are found in the LPS core oligosaccharides of most gram-negative bacteria (260), but only a few bacterial species (including *H. pylori*) can incorporate D-glycero-D-manno-heptose (DD-Hep) (250, 251). *H. pylori* incorporates both LD- and DD-heptose residues into its LPS structures (251). In a study analyzing LPS glycosyltransferase genes in a collection of *H. pylori* genomes, a high proportion of *H. pylori* strains classified as hpEurope (46 of 59, 78%) were found to possess a gene encoding a specific heptan transferase (corresponding to HP1283 in prototype strain 26695) that facilitates the incorporation of heptan (DD-Hep polymers) into the LPS O-antigen structure (250). Many of the HP1283-positive strains also contained a gene (HP1578) encoding a GlcNAc transferase that initiates Lewis antigen synthesis onto the heptan moiety (250) (Fig. 6). In contrast, HP1283 and HP1578 genes were absent from all 74 East Asian strains analyzed, suggesting that these strains fail to incorporate heptan into the LPS O-antigen region. Conversely, the East Asian strains possess two copies of HP1105 (a β-1,3-GlcNAc transferase) and JHP0562 (a β-1,3-Gal transferase), which enable Lewis antigen synthesis in East Asian strains (250, 251). hpEurope strains have only a single copy of HP1105 and lack JHP0562. The presence or absence of heptan in hpEurope LPS versus East Asian LPS could potentially influence interactions of the corresponding *H. pylori* strains with host cells. It has been proposed that differences in the heptan content of strains might contribute to immune evasion by influencing the exposure of *H. pylori* LPS Lewis antigens to immune cells (251).

Thus far, studies have focused on differences in LPS characteristics between hpEurope *H. pylori* strains and East Asian strains. An initial genetic analysis showed that HP1283 was absent not only in East Asian strains, but also commonly absent in strains from other geographic regions, including strains classified as hpAfrica1, hpAsia2, and Indigenous American (250). Further analysis of the prevalence of LPS glycosyltransferase genes from a larger number of strains from diverse geographic locations will be needed to better understand how variations in *H. pylori* LPS structure influence gastric cancer risk.

ADP-Hep, a precursor for the incorporation of LD-Hep and DD-Hep into *H. pylori* LPS, is a pathogen-associated molecular pattern (PAMP) that can activate proinflammatory host responses. In *H. pylori*, ADP-Hep is an LPS metabolite delivered into host cells through a Cag T4SS-dependent process, leading to activation of the ALPK1-TIFA signaling pathway and activation of NF-κB (108, 109, 251). It has been proposed that the inability of East Asian *H. pylori* strains to incorporate heptan into the LPS O-antigen region may influence the levels of free LPS metabolites (such as ADP-Hep) that are available for release into host cells (251). Further studies are required to investigate how differences in the LPS structure of Western and East Asian strains, as well as strains from other geographic regions, might impact *H. pylori*-host interactions, host inflammatory responses, and gastric cancer risk.

## NON-RANDOM DISTRIBUTION OF *H. PYLORI* GENETIC VARIATIONS THAT INFLUENCE GASTRIC CANCER RISK

As described in the previous sections, *H. pylori* strains containing the *cag* PAI, s1/i1/m1 *vacA* allelic types, and specific OMP profiles are associated with an increased risk of gastric cancer or peptic ulcer disease, compared to *H. pylori* strains lacking these features (Table 1). Moreover, a high proportion of *H. pylori* strains isolated in East Asia, which has a high incidence of gastric cancer, contain the *cag* PAI, type s1 *vacA*, and the OMP characteristics listed in Table 1 (91, 126, 131, 216). In Western countries, there is a non-random distribution of these genetic variations in *H. pylori* strains. Strains containing the *cag* PAI typically contain s1 *vacA* alleles instead of s2 *vacA* alleles, and strains containing s2 *vacA* alleles typically lack *cagA* (127, 184). Strains containing J-Western forms of *cagA* typically contain m2 forms of *vacA* (153, 261). Strains containing the *cag* PAI and s1 *vacA* alleles commonly contain *babA* (encoding the BabA outer membrane protein adhesin) (198, 211, 216, 232), *homB* (198, 216, 232, 238–240), type I *hopQ* alleles (225), and intact *oipA* alleles (232, 235). Conversely, strains lacking the *cag* PAI typically lack these OMP genetic features. *H. pylori* undergoes intergenomic recombination at a high frequency (63, 64). Therefore, the non-random distribution of these strain-specific genetic features probably reflects a selective advantage conferred by their mutual presence or absence (262–264).

Because of the non-random distribution of the genetic variations shown in Table 1, it is challenging to determine the extent to which variations in the individual genes influence gastric cancer risk, based solely on epidemiologic studies. However, several studies have reported that i1 or m1 forms of *vacA* are associated with an increased risk of intestinal metaplasia, gastric epithelial damage, or peptic ulcer disease (compared to i2 or m2 forms of *vacA*), after controlling for *cagA* status (124, 187, 195).

## *H. PYLORI*–HUMAN CO-EVOLUTION

### Optimization of *H. pylori* fitness

Non-synonymous mutations in many genes encoding proteins involved in *H. pylori*–host interactions (e.g., *cagA*, *cag* PAI genes encoding components of the Cag type IV secretion system, *vacA*, and *babA*) exhibit signatures of diversifying selection or fixation in specific human populations, as described in previous sections (86, 89–91, 153, 181, 183, 265–268). As expected, genes encoding proteins with roles in *H. pylori*–host interactions typically exhibit higher levels of positive selection than housekeeping genes (86, 89–91, 153, 181, 183, 265, 266, 268). The observed geographic differences in sequences of genes involved in *H. pylori*–host interaction likely reflect key roles of these genes in *H. pylori* adaptation to specific human populations. Positively selected mutations might confer increased bacterial fitness by enhancing the ability of strains to transmit to new hosts, colonize new hosts, persist in the stomach, or compete with other *H. pylori* strains (or other bacterial species) within the human stomach. While all positively selected changes are presumed to enhance bacterial fitness, only a small subset of the positively selected changes are likely to be relevant for gastric cancer pathogenesis. The functional consequences of most of the positively selected changes have not yet been evaluated experimentally.

Relatively little is known about the selective forces that drive *H. pylori* genetic diversification or how selective forces might vary among different geographic regions. Genome sequence analyses have shown that *H. pylori* undergoes a rapid mutational burst upon colonization of new hosts, which suggests that transmission to new hosts is associated with strong selective pressures (65). Varying properties of receptors for *H. pylori* adhesins among individual humans might be a selective force (209). Experiments in animal models have shown that gastric inflammation and dietary content (including salt or iron content) can also be strong selective pressures (269–272). Geographic variation in host receptors for *H. pylori* proteins and geographic variations in diet may drive selection of *H. pylori* variants that have region-specific fitness advantages.

In mouse and rhesus macaque models, *H. pylori* strains commonly lose Cag T4SS activity over time due to acquisition of mutations in the *cag* PAI that disrupt T4SS function (273, 274). A similar phenomenon has been observed with BabA in the mouse model (275). Therefore, *cag* PAI-negative strains and *babA*-negative strains have a selective advantage compared to *cag* PAI-positive/*babA*-positive strains in the mouse model (273, 275, 276). A recent study sought to identify physiologically relevant selective pressures that promoted maintenance of Cag T4SS activity in the mouse model (277). The loss of T4SS activity was blunted by two conditions: dietary iron restriction and systemic *Salmonella* coinfection. Retention of Cag T4SS activity under conditions of dietary iron restriction might be attributable to a role of the Cag T4SS in acquisition of iron from the host (277). Systemic Salmonella infection might promote retention of Cag T4SS activity through multiple mechanisms, including iron sequestration and a generalized increase in inflammatory processes associated with systemic infection. These findings illustrate how strain-specific genes and environmental exposures can influence *H. pylori* fitness.

Since the *cag* PAI and *babA* are commonly present in *H. pylori* isolates from humans (275, 276); these genes presumably provide fitness advantages to *H. pylori* under certain conditions in the human gastric environment. The *cag* PAI is a large genetic element (40 kb), and assembly of the Cag T4SS is an energetically costly process. Retention of an intact *cag* PAI in a high proportion of *H. pylori* strains would not be beneficial unless it conferred a selective advantage. Most *H. pylori* strains isolated in East Asia contain an intact *cag* PAI, but *cag* PAI-negative strains are commonly isolated in other parts of the world (46, 119, 134). Several studies have suggested that *cag* PAI-positive strains are disappearing more rapidly than *cag* PAI-negative strains in some parts of the world (278, 279), and one recent study demonstrated that a lineage of *cag* PAI-negative strains has rapidly expanded in the United States over the past 200 years (46). The expansion of a *cag* PAI-negative lineage suggests that it has a high level of fitness in the United States. We speculate that recent changes in the proportion of *H. pylori* strains containing the *cag* PAI are attributable to recent changes in selective pressures. One study reported that *cag* PAI-positive strains may be more susceptible to antibiotic treatment than *cag* PAI-negative strains (280). Therefore, we speculate that increasing rates of antibiotic use, as well as changes in diet and a decreasing incidence of other gastrointestinal infections (277), may favor the proliferation of *cag* PAI-negative strains in some geographic regions.

### Impact of prolonged *H. pylori*–human co-evolution on gastric disease

*H. pylori* has been coevolving with human hosts for more than 100,000 years (75, 281). The potential consequences of prolonged *H. pylori*–human coevolution are exemplified by *H. pylori*–host interactions in Africa. Early studies noted that although *H. pylori* colonization of the stomach was common in African populations, these populations had a low rate of peptic ulcer disease and gastric cancer. This phenomenon was termed “the African enigma” (282). A similar association of African *H. pylori* strains with a low rate of gastric cancer has been noted in other parts of the world (283, 284). One study investigated whether the ancestral origins of *H. pylori* strains were associated with gastric cancer risk in 1,220 subjects from Venezuela, Colombia, Mexico, and Paraguay who were infected with *cagA*-positive *H. pylori* strains (285). Fifty genetic variations mapping to the *cag* PAI were used as the basis for determining the phylogenetic origin of the strains. This study reported an inverse association between *H. pylori* African ancestry and the risk of gastric cancer (285).

Thus far, there has been relatively little investigation of the features of African *H. pylori* strains that mediate bacteria–host interactions. HpAfrica2 strains do not contain the *cag* PAI (47, 117), but the *cag* PAI is commonly present in other populations of African strains (91, 132) (Fig. 2). One study analyzed six *H*. *pylori* strains isolated from patients in Colombia, which were classified as having predominantly European ancestry or predominantly African ancestry (286). The European strains were associated with more severe gastric histologic changes than the African strains (286). The strains were co-cultured with gastric epithelial cells, and *H. pylori* gene expression was analyzed using microarray methodology (286). The analysis identified 496 differentially expressed *H. pylori* genes (146 exhibiting increased expression in European strains and 350 exhibiting increased expression in African strains). Although all six strains contained *cagA* and s1m1 *vacA*, the European strains expressed these two genes at relatively higher levels compared to the other strains. The strains also elicited different responses in AGS cells, which were derived from a Caucasian person of both European and Indigenous American ancestry (287). The European strains induced higher IL-8 expression than the African strains, and the African strains induced increased apoptosis compared to the European strains (286). Another study showed that *cagA*-positive *H. pylori* strains from the Andean region of Colombia (high gastric cancer risk) induced higher expression of spermine oxidase (which generates hydrogen peroxide), increased oxidative DNA damage, and less apoptosis in gastric epithelial cells, compared to *cagA*-positive strains (predominantly African origin) from the coastal region of Colombia (lower gastric cancer risk) (288). Collectively, these studies suggest that the phylogeographic origin of *H. pylori* strains can influence interactions with gastric epithelial cells and suggest that strains with African ancestry may be less virulent than strains with European ancestry. Additional studies, using larger numbers of strains, will be required to further evaluate this hypothesis.

### Recent changes in *H. pylori*–human relationships

Human migrations over long distances have occurred for at least 60,000 years (78, 79), but for most of human history, global movements of human populations have been restricted by impediments to traveling long distances. Consequently, *H. pylori* and human populations have co-evolved together in many different parts of the world. Over the past 500 years, the development of global transportation systems has resulted in frequent intermingling of human populations from diverse geographic origins. When populations of diverse geographic origins intersect, what are the consequences for *H. pylori* and human health?

Several studies have investigated this topic in regions of South America or Central America, where multiple human populations (Indigenous American, European, and African origin) have intersected over the past 500 years (86, 90, 283, 284, 289, 290). Each of these human populations originally carried distinctive *H. pylori* strains. When present together in the same geographic regions, the *H. pylori* strains from different parts of the world presumably competed. *H. pylori* strains of Indigenous American origin can still be isolated in the Americas, but these strains are mainly found in Indigenous American human populations living in remote locations (291), where they have had minimal contact with European or African populations. The relative paucity of Indigenous American *H. pylori* strains in the Americas today, despite continued Indigenous American genetic contributions to current human populations (284), suggests that Indigenous American *H. pylori* strains were outcompeted by European and African strains.

Admixture of *H. pylori* strains from different geographic origins can lead to the emergence of hybrid or mosaic strains that arise through recombination, sometimes resulting in variants with favorable properties (79, 86, 90, 292). Consistent with this viewpoint, whole-genome sequence analysis of *H. pylori* strains isolated in the Americas revealed that most strains have genetic characteristics reflecting an origin from multiple ancestral *H. pylori* populations (290, 292). Among the American strains analyzed in one study, many of the genes exhibiting a high fixation index (Fst value), including *cagA* and other *cag* PAI genes, *vacA*, and *babA,* have roles in *H. pylori*-host interactions, suggesting that variation in these genes facilitates *H. pylori* adaptation to specific human populations (292).

Admixture of human populations and *H. pylori* of varying origins can potentially have an impact on human health and disease. This topic was evaluated by a study conducted in Colombia, where the human populations and *H. pylori* strains have multiple ancestral origins (284). The study analyzed patients from two different regions of Colombia (the mountains and the coast). The two populations had similar rates of *H. pylori* colonization but different incidences of gastric cancer. The population in the mountainous region (Tuquerres) had an incidence of gastric cancer that was about 25-fold higher than that of the population in a coastal region (Tumaco). The genetic ancestry of the *H. pylori* strains and corresponding human hosts was then characterized. The high-risk human population in the mountainous region had mostly Indigenous American ancestry and was colonized predominantly by European strains of *H. pylori*. In contrast, the low-risk human population in the coastal regions had predominantly African ancestry and was colonized predominantly by African strains of *H. pylori. H. pylori* strains of predominantly Indigenous American ancestry were not identified in either population. Notably, African *H. pylori* strains had a deleterious effect on gastric pathology in the mountainous region, suggesting that an *H. pylori*–host mismatch, not the strain itself, was responsible for the increased risk of gastric disease. Mismatched ancestry was a stronger indicator of gastric disease risk than the presence of *cagA* (284). The results of this study suggest that admixture of populations can potentially have an impact on the severity of gastric disease.

To further investigate this hypothesis, one study investigated the gene expression profiles of several human gastric cell lines co-cultured with *H. pylori* strains. The study tested three human gastric cell lines with varying ancestral origins (predominantly European [Hs746T cells], predominantly African [NCI-N87], or predominantly East Asian [MKN74]) and two *H*. *pylori* strains (26695 and J99, predominantly European or predominantly African origin, respectively), which are both *cagA*-positive and contain s1m1 forms of *vacA* (293). As expected, substantial differences in gene expression profiles were noted among the three cell lines. In response to *H. pylori*, the African cell line primarily upregulated lipid metabolism, the East Asian cell line primarily activated energy production, and the European cell line primarily upregulated immune-related genes. The African cell line had a decreased pro-inflammatory response and a more effective response to oxidative stress compared to the European cell line (293). The two *H*. *pylori* strains induced similar changes in the gastric cell lines. Further studies will be required to evaluate the biological relevance of these observations to our understanding of the consequences of mismatch between the ancestries of *H. pylori* strains and human populations.

## TOPICS FOR FURTHER RESEARCH

Over the past few decades, numerous studies have characterized the global geographic diversity that exists among *H. pylori* strains. As described in this review, these genetic variations are important factors contributing to geographic variations in gastric cancer rates. Additional factors are certainly relevant, including geographic differences in diet, environmental exposures, and human genetic variations.

There are many limitations of the analyses of *H. pylori* geographic diversity and gastric cancer risk that have been conducted thus far. Most of the analyses have focused on a small number of *H. pylori* genes known to encode proteins that interact with host cells, including secreted proteins and outer membrane proteins. Additional genetic variations are likely to influence *H. pylori*–host interactions and disease outcomes. Moreover, some genetic variations that are known to affect *H. pylori*–host interactions or gastric cancer risk (e.g., sequence variation in *htrA*, encoding a serine protease) (294) have not been investigated in detail to evaluate possible geographic variation. In future studies, it will be important to employ systematic whole genome sequencing methods to analyze a broader array of genes, define how variations in those genes correlate with disease status, and evaluate potential geographic variations in the corresponding genetic features. Analyses of geographic variations in *H. pylori* transcriptional profiles, restriction enzyme profiles, epigenomic profiles, and post-translational changes will also be informative.

Many of the analyses conducted thus far have compared genetic features of East Asian *H. pylori* strains to Western *H. pylori* strains. This has been a logical approach, predicated on the high gastric cancer rates in many East Asian countries compared to Western countries. In future studies, it will be important to analyze and compare *H. pylori* strains isolated in other geographic regions. Such studies may provide new insights into the varying rates of gastric cancer throughout the world.

In conjunction with epidemiologic studies, it will also be important to undertake *in vitro* experiments to analyze how geographic variations among strains impact *H. pylori* biology and the functions of specific *H. pylori* proteins. Further studies of positively selected genetic variations in cell culture models or biochemical assays will provide insight into whether specific changes confer phenotypic alterations relevant to gastric pathogenesis, and it will be informative to analyze the locations of corresponding amino acids within *H. pylori* protein structures. Experiments in animal models may also be useful, but there are limitations in the use of current rodent models for assessing the activities of *H. pylori* constituents that have evolved for optimal function in humans (222, 295).

Finally, it will be important to investigate further how recent changes in human behavior are impacting *H. pylori* and gastric diseases linked to *H. pylori*. It will be important to understand how changes in diet, food storage, improved sanitation, and antibiotic use are influencing the prevalence of *H. pylori*, *H. pylori*-associated gastric diseases, extra-gastric diseases inversely associated with *H. pylori* (2, 281, 296, 297), and *H. pylori* evolution. Further studies are also needed to assess how recent increases in human geographic mobility are affecting *H. pylori* and human health. Admixture of *H. pylori* strains from different parts of the world results in competition among strains that have evolved in relative isolation over thousands of years, as well as opportunities for recombination among strains from different geographic origins. These circumstances are likely having a sizable impact on the evolution of *H. pylori* as a species, and disruptions in coevolved *H. pylori*–human relationships are likely influencing the incidence or severity of diseases linked to *H. pylori*, including gastric cancer.
